# Dephosphorylated Polymerase I and Transcript Release Factor Prevents Allergic Asthma Exacerbations by Limiting IL-33 Release

**DOI:** 10.3389/fimmu.2018.01422

**Published:** 2018-06-21

**Authors:** Yingmeng Ni, Jimin Hao, Xiaoxia Hou, Wei Du, Youchao Yu, Tiantian Chen, Zhuang Wei, Yangyang Li, Fuxiang Zhu, Shuaiwei Wang, Rui Liang, Dan Li, Yue Lu, Kan Liao, Bin Li, Guochao Shi

**Affiliations:** ^1^Department of Pulmonary and Critical Care Medicine, Rui Jin Hospital, Shanghai Jiao Tong University School of Medicine, Shanghai, China; ^2^Institute of Respiratory Diseases, Shanghai Jiao Tong University School of Medicine, Shanghai, China; ^3^Laboratory of Cell Biology, Institute of Biochemistry and Cell Biology, Shanghai Institutes for Biological Sciences, Chinese Academy of Sciences, Shanghai, China; ^4^Department of Immunology and Microbiology, Shanghai Institute of Immunology, Shanghai Jiao Tong University School of Medicine, Shanghai Jiao Tong University, Shanghai, China; ^5^School of Pharmacy, Shanghai University of Traditional Chinese Medicine, Shanghai, China

**Keywords:** IL33, PTRF, allergic asthma, asthma exacerbation, airway epithelial cells

## Abstract

**Background:**

Asthma is a chronic inflammatory disease characterized by airway inflammation and airway hyperresponsiveness (AHR). IL-33 is considered as one of the most critical molecules in asthma pathogenesis. IL-33 is stored in nucleus and passively released during necrosis. But little is known about whether living cells can release IL-33 and how this process is regulated.

**Objective:**

We sought to investigate the role of polymerase I and transcript release factor (PTRF) in IL-33 release and asthma pathogenesis.

**Methods:**

Ovalbumin (OVA)-induced asthma model in PTRF^+/−^ mice were employed to dissect the role of PTRF *in vivo*. Then, further *in vitro* experiments were carried out to unwind the potential mechanism involved.

**Results:**

In OVA asthma model with challenge phase, PTRF^+/−^ mice showed a greater airway hyper-reaction, with an intense airway inflammation and more eosinophils in bronchoalveolar lavage fluid (BALF). Consistently, more acute type 2 immune response in lung and a higher IL-33 level in BALF were found in PTRF^+/−^ mice. In OVA asthma model without challenge phase, airway inflammation and local type 2 immune responses were comparable between control mice and PTRF^+/−^ mice. Knockdown of PTRF in 16HBE led to a significantly increased level of IL-33 in cell culture supernatants in response to LPS or HDM. Immunoprecipitation assay clarified Y158 as the major phosphorylation site of PTRF, which was also critical for the interaction of IL-33 and PTRF. Overexpression of dephosphorylated mutant Y158F of PTRF sequestered IL-33 in nucleus together with PTRF and limited IL-33 extracellular secretion.

**Conclusion:**

Partial loss of PTRF led to a greater AHR and potent type 2 immune responses during challenge phase of asthma model, without influencing the sensitization phase. PTRF phosphorylation status determined subcellular location of PTRF and, therefore, regulated IL-33 release.

## Introduction

Asthma is the most common non-communicable respiratory disease, affecting ~300 million people globally (1). It is characterized by chronic airway inflammation, usually of allergic nature, leading to recurrent episodes of wheezing, coughing, and shortness of breath. Despite significant advances in asthma management, acute exacerbations continue to occur and still account for the majority of morbidity, mortality, and costs of asthma (2).

The IL-33/ST2-ILC2 axis plays a crucial role in asthma. The genes encoding IL-33 and ST2/IL1RL1 have been identified as major susceptibility loci for human asthma in several genome-wide association studies. Moreover, in all these studies, which included thousands of patients from diverse ethnic groups and different forms of asthma, IL33 and ST2/ILRL1 were the only two genes reproducibly found to be associated with asthma (3).

Abundant expression of the endogenous IL-33 protein has been observed in epithelial cells from tissues exposed to the environment, including airway epithelium. Full-length IL-33 is stored in the nucleus and released as an alarmin from cells dying after injury or by necrosis (4). Different proteases can process the biologically active full-length IL-33 in its N terminus, yielding shorter forms that show enhanced biological activity compared to full-length IL-33. However, when the core IL-1 structure in its C terminus is altered, bioactivity is lost. During apoptosis, full-length IL-33 is cleaved by apoptotic caspase-3 and caspase-7 and inactivated (5, 6).

Cell death by injury or necrosis should not be the only mechanism by which “homeostatic” IL-33 can be released from cells. Previous studies show that mechanical stress results in the release of bioactive IL-33 by cardiac endothelial cells and fibroblasts without cell death, and cardiac endothelial cells actively releases IL-33 resulting subsequent systemic inflammation in mice hypertension model (7). Several types of cells, including Clara cell, epithelial progenitor cells, and corneal epithelial cells, were found to be able to release IL-33 on response of ATP (8, 9). However, since IL-33 lacks a signal sequence, it is not likely to be secreted *via* the endoplasmic reticulum–Golgi secretory pathway (10). The molecular mechanisms of release are not yet clear.

To explore the potential underlying mechanism of controlled release of IL-33, we performed a tandem affinity purification (TAP) of IL-33 protein. High-performance liquid chromatography-mass spectrometry of IL-33 protein complex showed an interaction between IL-33 and polymerase I and transcript release factor (PTRF), which is further confirmed by co-immunoprecipitation (Figure S1 in Supplementary Material).

Polymerase I and transcript release factor, also known as Cavin-1, was first described to play a role in the termination of transcription (11). More recently, it is shown that PTRF is also essential in the formation of caveolae (12). Lung tissue high express PTRF, and type I epithelial cells and endothelial cells demonstrate numerous caveolae (13). PTRF knockout mice have altered lung physiology, evidenced by increased airway resistance and lung elastance. Altered lung morphology has been reported in PTRF knockout mice, including interstitial thickening and hypercellularity with an increased collagen deposition in lungs. These physiological and morphological changes were companied, with an excessive recruitment of CD45^+^ cells and macrophages (14).

To illustrate the role of PTRF in IL-33 release and asthma development, we use PTRF^+/−^ mice to show that loss of PTRF led to a greater airway hyper-reaction, with an intense airway inflammation and potent type 2 immune responses. Knockdown of PTRF in 16HBE causes an excessive release of IL-33 after LPS and HDM treatment. The dephosphorylated mutant of PTRF shows an increased location in nucleus and prevents the release of IL-33. Taken together, our results show that dephosphorylated PTRF prevents allergic asthma exacerbations by limiting IL-33 release.

## Results

### Partial Loss of PTRF Leads to Excessive Eosinophilic Airway Inflammation

**Figure 1:**
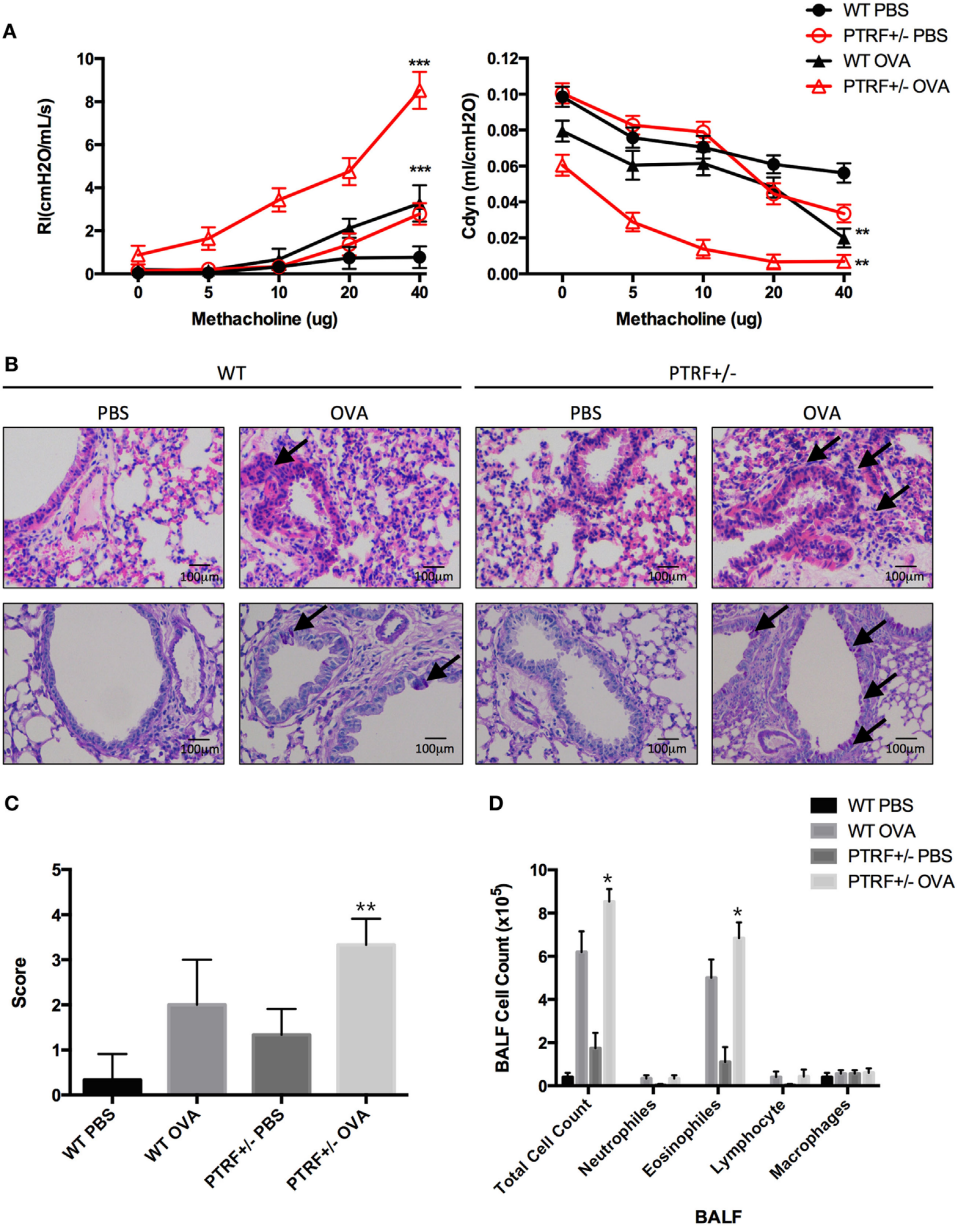
Partial loss of polymerase I and transcript release factor (PTRF) led to airway hyperresponsiveness (AHR) and eosinophilic airway inflammation. Mice were intraperitoneally injected with phosphate-buffered saline (PBS) or ovalbumin (OVA) on days 0, 7, and 14, and then challenged with PBS or OVA on days 21–27. **(A)** AHR in response to inhaled methacholine was measured in PBS- or OVA-challenged mice. Maximal responses to increasing doses of methacholine are shown for lung resistance (Rl) and dynamic compliance (Cdyn). **(B)** Representative images of hematoxylin and eosin staining of lung tissue (upper panel), and periodic acid-schiff stain (lower panel). **(C)** Inflammation scores of lung tissues. **(D)** Cell classification in bronchoalveolar lavage fluid. *p < 0.05, **p < 0.01, ***p < 0.001. Results are pooled data from four independent experiments (mean ± SEM of *n* = 3–4 mice in each group).

To illustrate the function of PTRF in asthma development, we carried out ovalbumin (OVA)-induced asthma model in PTRF^+/−^ mice. Mice were injected intraperitoneally with phosphate-buffered saline (PBS) or OVA on day 1, day 7, and day 14, then challenged with PBS or OVA for 7 days continuously. Since asthma is characterized by airway hyperresponsiveness (AHR), we first investigated AHR in the OVA-induced mouse asthma model. Lung resistance and dynamic compliance, in response to aerosolized methacholine, were determined. PTRF^+/−^ mice showed an increased AHR in all parameters measured, compared with their wide-type littermates (Figure 1A).

Lung specimens and bronchoalveolar lavage fluid (BALF) were collected 24 h after the last challenge. HE staining and PAS staining showed that OVA-treated PTRF^+/−^ mice suffered from an increased lymphocyte infiltration around airway, an excessive mucus production (Figure 1B), and a more severe lung inflammation (Figure 1C), compared with their wide-type littermates. And this more pronounced lung inflammation was accompanied with an increased account of eosinophils in BALF (Figure 1D).

### Partial Loss of PTRF Leads to Enhanced Local Type 2 Immune Responses

**Figure 2:**
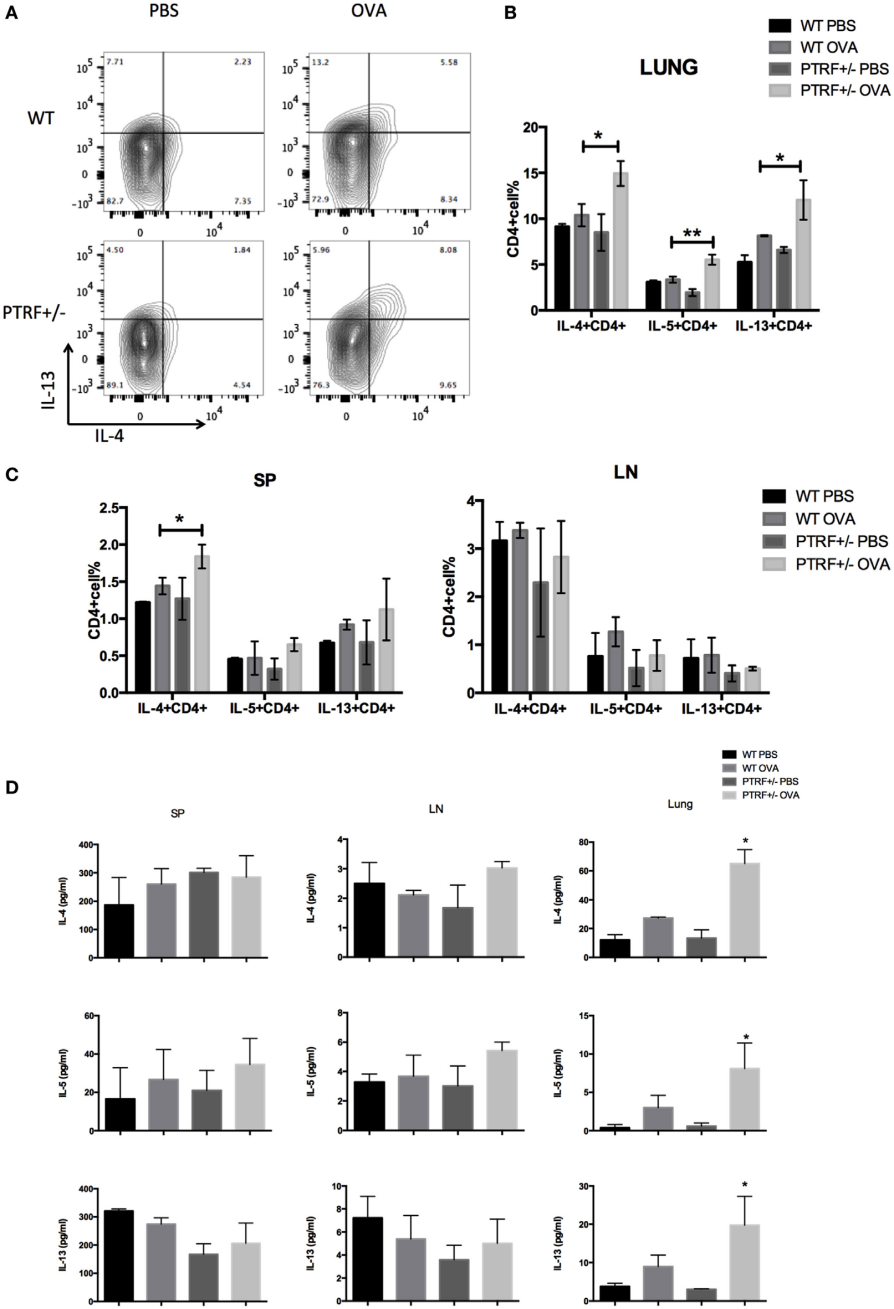
Partial loss of polymerase I and transcript release factor (PTRF) led to enhanced local type 2 immune responses. Mice were intraperitoneally injected with PBS or ovalbumin (OVA) on days 0, 7, and 14, and then challenged with PBS or OVA on days 21–27. **(A)** Representative figure shown the IL-13^+^IL-4^+^CD4^+^T cells from WT and PTRF^+/−^ littermates. **(B)** Percentages of IL-4^+^, IL-5^+^, IL-13^+^ CD4^+^ T cells from lung of WT and PTRF^+/−^ littermates. **(C)** Percentages of IL-4^+^, IL-5^+^, IL-13^+^ CD4^+^ T cells from spleen and peripheral lymph nodes of WT and PTRF^+/−^ littermates. **(D)** IL-4, IL-5, and IL-13 levels in culture supernatant of lymphocytes from lung, spleen, and peripheral lymph nodes of WT and PTRF^+/−^ littermates. *p < 0.05, **p < 0.01, as determined by Student’s *t*-test. Results are pooled data from four independent experiments (mean ± SEM of *n* = 4 mice in each group).

Type 2 immune responses are the core of OVA-induced asthma model. Thus, we further analyzed type 2 cytokines by intracellular staining. We observed excessive local Th2 responses in OVA-treated PTRF^+/−^ mice, where CD4^+^ T cells isolated from lungs produced higher levels of IL-4, IL-5, and IL-13 on *in vitro* stimulation (Figures 2A,B). However, CD4^+^ T cells from spleen and peripheral lymph nodes produced similar levels of type 2 cytokines in all groups, except that the percentage of IL-4^+^CD4^+^ T cells slightly increased in splenic lymphocytes of OVA-treated PTRF^+/−^ mice (Figure 2C).

Meanwhile, we used ELISA to analyze the levels of type 2 cytokines in culture supernatants of lymphocytes from spleen, peripheral lymph nodes, and lungs. Figure 2D showed a marked increased level of IL-4, IL-5, and IL-13 on *in vitro* stimulation in supernatants of lymphocytes from lungs of OVA-treated PTRF^+/−^ mice, compared with these of OVA-treated wide-type mice. But no difference of cytokine levels was observed in supernatants of lymphocytes from spleen and peripheral lymph nodes. These results indicate that PTRF has a greater effect on local immune responses than systemic immune responses.

### Partial Loss of PTRF Does Not Affect Airway Inflammation During Sensitization Phase

To further confirm whether PTRF plays roles in sensitization phase or challenge phase of OVA-induced mice asthma model, we carried out an asthma model only with the sensitization phase. Mice were injected intraperitoneally with PBS or OVA on day 1, day 7, and day 14, and then sacrificed on day 15. HE staining and PAS staining of lung tissues showed no airway inflammation or mucus production in OVA-treated wide-type mice or PTRF^+/−^ mice (Figure S2A in Supplementary Material). Consistently, intracellular staining of type 2 cytokines in lymphocytes from spleen, peripheral lymph nodes, and lungs did not show activation of type 2 immune responses, neither systemic nor local (Figure S2B in Supplementary Material). Levels of IL-4, IL-5, and IL-13 in culture supernatants of lymphocytes were also similar among four groups (Figure S2C in Supplementary Material). These results indicate that partial loss of PTRF only affect airway inflammation during asthma exacerbation, but not during the sensitization phase.

### PTRF Limits IL-33 Release

**Figure 3:**
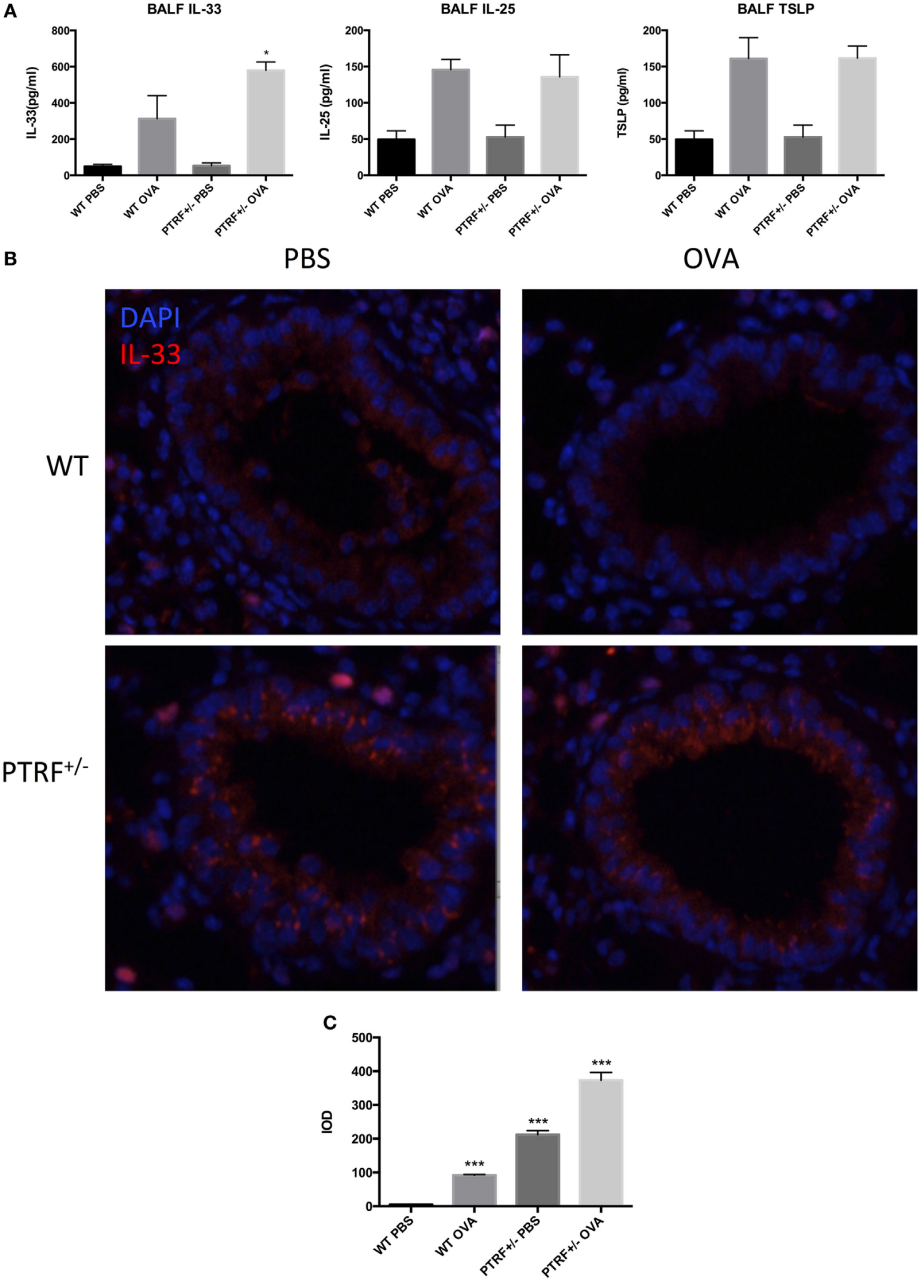
IL-33 release in airway. **(A)** IL-33, IL-25, and TSLP levels in bronchoalveolar lavage fluid. **(B)** Representative images of IL-33 (red) immunofluorescence of airway. **(C)** Integrated option density (IOD) of IL-33 (red) immunofluorescence images. *p < 0.05, ***p < 0.001, as determined by Student’s *t*-test. Results are pooled data from four independent experiments (mean ± SEM of *n* = 4 mice in each group).

Release of epithelial cytokines, IL-33, IL-25, and TSLP, is the initial process during asthma exacerbation. Thus, we collected BALF 24 h after the last challenge and check the levels of IL-33, IL-25, and TSLP in BALF. After OVA challenge, the levels of IL-33, IL-25, and TSLP in BALF were all significantly increased in wide-type mice or PTRF^+/−^ mice. The partial loss of PTRF only led to an excessive release of IL-33 in BALF, but not IL-25 or TSLP (Figure 3A). Moreover, the immunofluorescence of IL-33 in airway showed great increase in quantity of IL-33 in PTRF^+/−^ mice, regardless of whether the mice were treated with OVA or not (Figures 3B,C).

**Figure 4:**
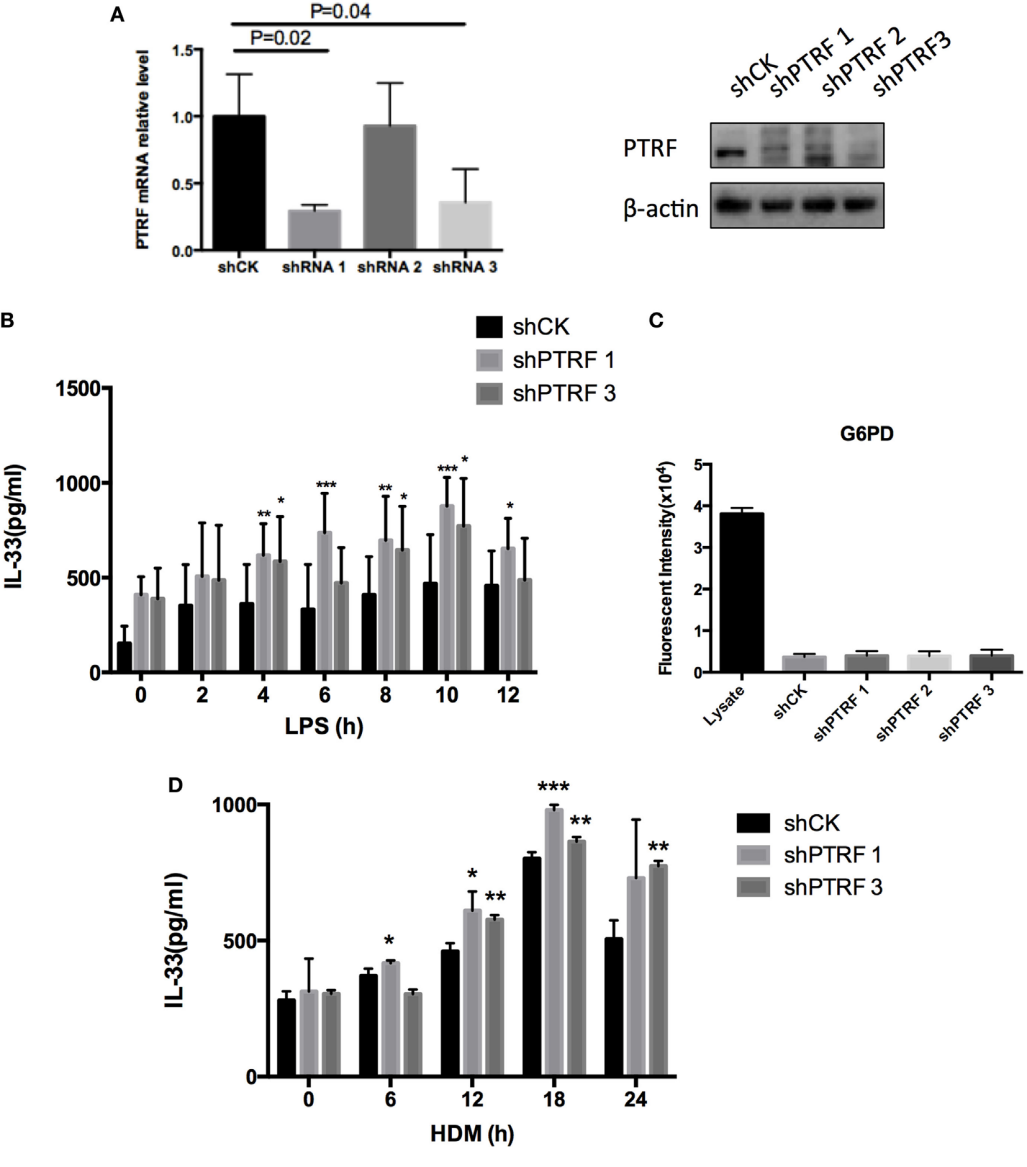
Knockdown of polymerase I and transcript release factor (PTRF) induced excessive IL-33 release. **(A)** Knockdown efficiency of shPTRF. mRNA level of PTRF (left panel) and protein level of PTRF (right panel). **(B)** IL-33 level in culture supernatant of HBE after LPS (1 ng/µl) treatment. **(C)** G6PD release assay in HBE with knockdown of shPTRF. **(D)** IL-33 level in culture supernatant of HBE after HDM (30 µg/µl) treatment. *p < 0.05, **p < 0.01, ***p < 0.001, as determined by Student’s *t*-test.

To confirm the effect of PTRF on IL-33 release *in vitro*, we knocked down PTRF by shRNAs in human bronchial epithelial cells (16HBE) (Figure 4A). After the mice were treated with LPS or HDM, levels of IL-33 in culture supernatants were checked. We observed a significant increase in release of IL-33 to supernatants on LPS or HDM stimulation when PTRF was depleted (Figures 4B,D). Moreover, this increased release of IL-33 was not due to cellular necrosis (Figure 4C) (15).

### PTRF Controls IL-33 Subcellular Location

**Figure 5:**
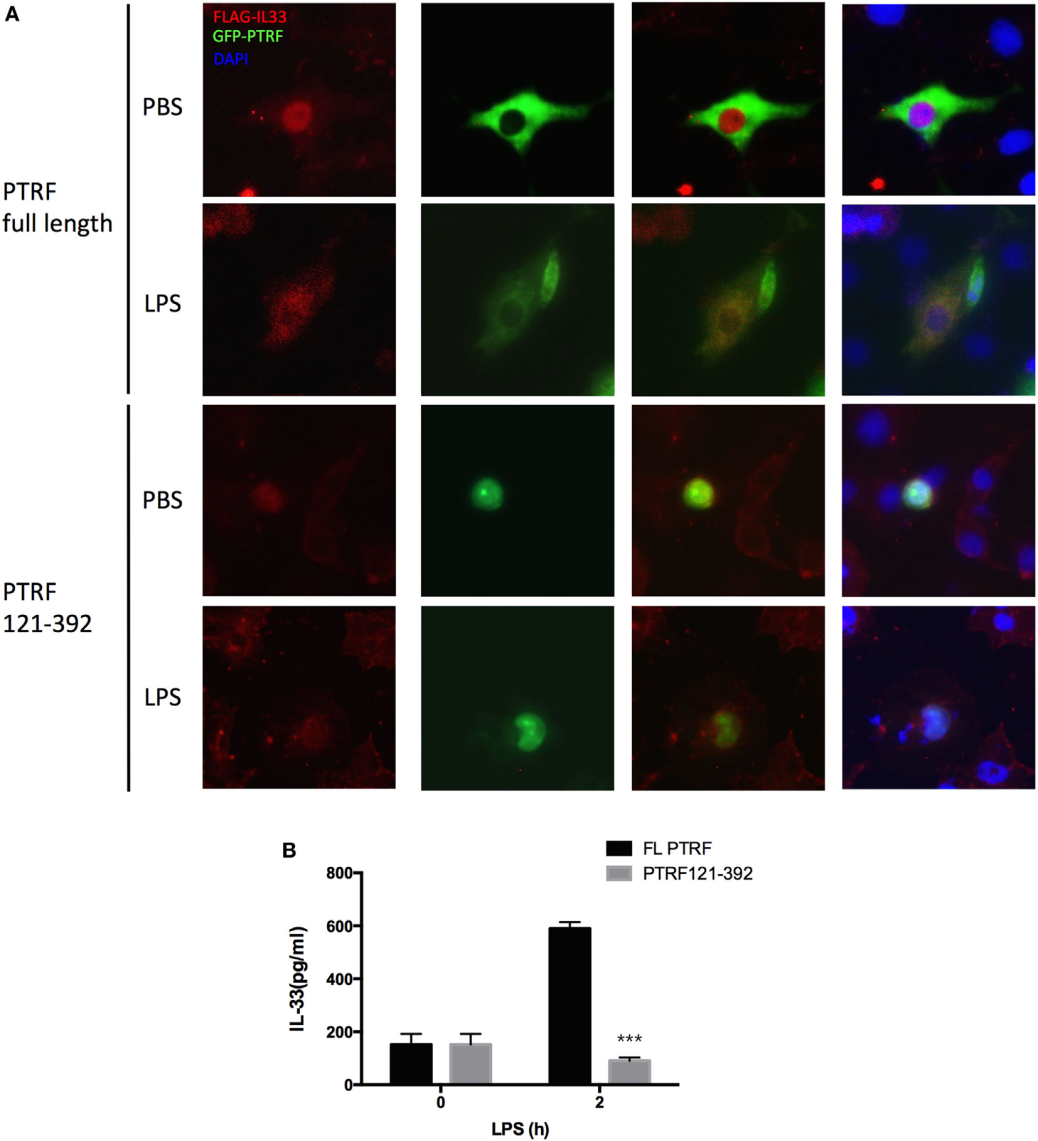
Polymerase I and transcript release factor (PTRF) controls IL-33 subcellular location. **(A)** Representative images of IL-33 (red) and PTRF (green) immunofluorescence of wide type PTRF or PTRF truncation 121-392 transfected HBE with or without LPS (1 ng/µl) treatment. **(B)** IL-33 levels in culture supernatants from wide type PTRF or PTRF truncation 121-392 transfected HBE with or without LPS (1 ng/µl) treatment.

The function of PTRF largely depends on its subcellular location (16). Meanwhile, IL-33 subcellular location is critical to its release process (15). Thus, we thought to check whether the subcellular locations of these two proteins affect each other. Interestingly, we observed that when a PTRF truncation 121-392, which keeps a nucleus location, was overexpressed in 16HBE, IL-33 was restricted in nucleus even on stimulation of LPS (Figure 5A). Consistently, the levels of IL-33 in culture supernatants were sharply decreased when PTRF 121-392 was overexpressed (Figure 5B).

### Phosphorylation of PTRF Plays a Key Role to Control IL-33 Release

**Figure 6:**
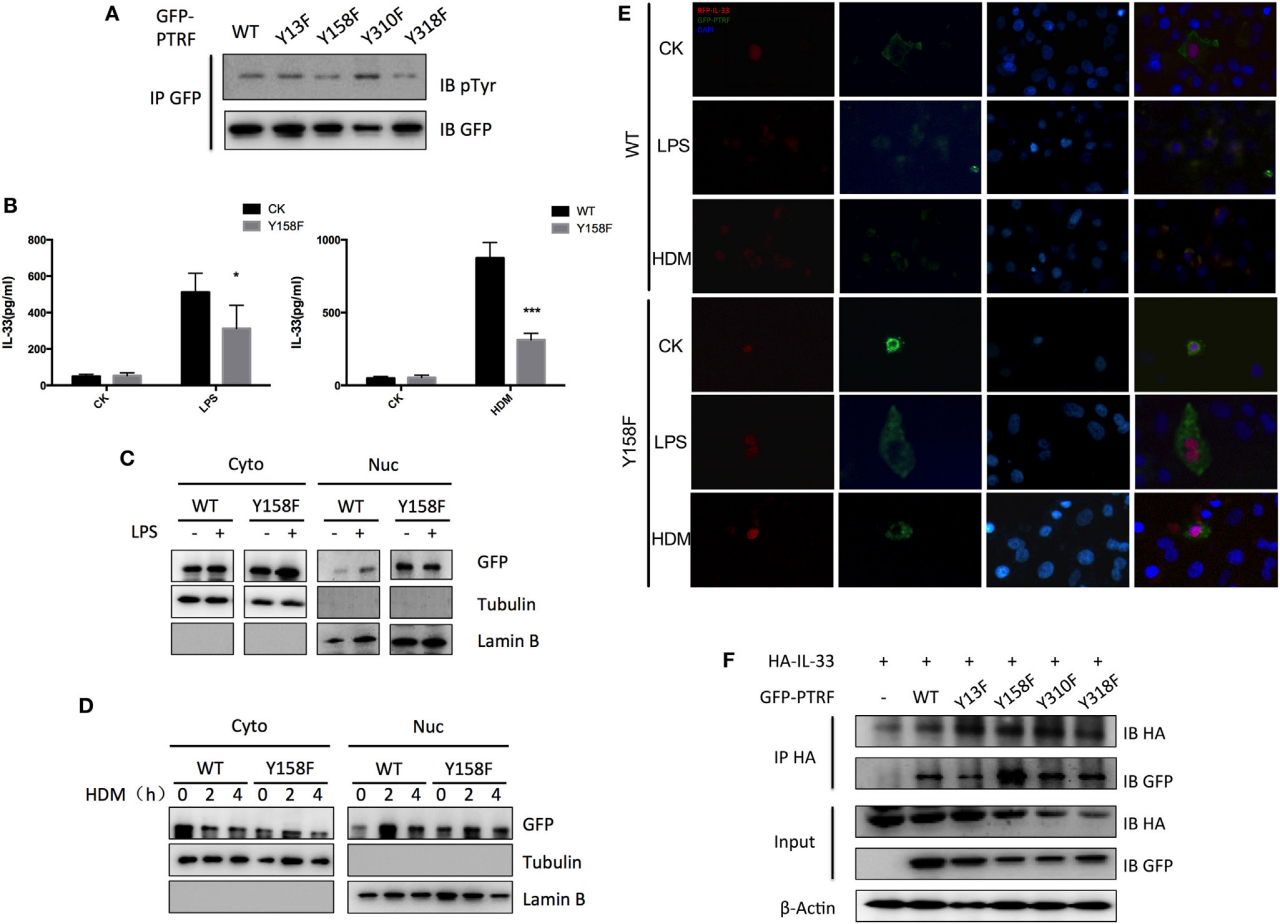
Phosphorylation of polymerase I and transcript release factor (PTRF) critically controls IL-33 release. **(A)** Whole cell lysates from wild type PTRF and various single tyrosine mutants (Y13F; Y158F; Y310F; Y318F) transfected HBE cell were immunoprecipitated with GFP antibody followed by SDS-PAGE and blotted with indicated antibodies. **(B)** IL-33 level of culture supernatant of wild type PTRF and Y158F mutated PTRF transfected HBE cell with or without LPS (1 ng/µl) or HDM (30 µg/µl) treatment. **(C)** Nuclear and cytoplasmic protein extraction from wild type PTRF and Y158F mutated PTRF transfected HBE cell with or without LPS (1 ng/µl) treatment were blotted with indicated antibodies. **(D)** Nuclear and cytoplasmic protein extraction from wild type PTRF and PTRF Y158F mutant transfected HBE cell with or without HDM (30 µg/µl) treatment were blotted with indicated antibodies. **(E)** Representative images of IL-33 (red) and PTRF (green) immunofluorescence of wide type PTRF or Y158F mutated PTRF transfected HBE with or without LPS (1 ng/µl) or HDM (30 µg/µl) treatment. **(F)** Whole cell lysates from wild type PTRF and Y158F mutated PTRF transfected HBE cell were immunoprecipitated with anti-HA antibody followed by SDS-PAGE and blotted with indicated antibodies. *p < 0.05, **p < 0.01, ***p < 0.001, as determined by Student’s *t*-test.

Protein modifications are often linked to its cellular localizations. PTRF can be phosphorylated or dephosphorylated at tyrosine sites by several extracellular signals. Next, we investigated whether phosphorylation of PTRF influences IL-33 release. Since PTRF has only four tyrosine sites, we substituted each of these with phenylalanine. Y158F showed a significant decrease in phosphotyrosine levels (Figure 6A). Over-expression of this mutant form (Y158F) in 16HBE led to decreased levels of IL-33 on stimulation of LPS or HDM (Figure 6B).

To investigate whether tyrosine phosphorylation of PTRF plays a role in subcellular location of PTRF itself and IL-33, cytoplasmic and nuclear fractionation and immunofluorescence were conducted. We observed that wide type PTRF mainly localized in cytoplasm and translocated into nucleus on stimulation of LPS and HDM (Figures 6C,D). However, quite a few PTRF Y158F mutant located into nucleus even without stimulation, and the translocation on stimulation was not as obvious as wide type PTRF (Figures 6C,D). Immunofluorescence assay showed that on stimulation of LPS or HDM, IL-33 and PTRF co-located in cytoplasm (Figure 6E, left panel). However, over-expression of PTRF Y158F mutant led to sequestration of IL-33 in nucleus (Figure 6E, right panel). Moreover, co-immunoprecipitation showed a significantly enhanced reaction of IL-33 with PTRF Y158F mutant (Figure 6F).

**Figure 7:**
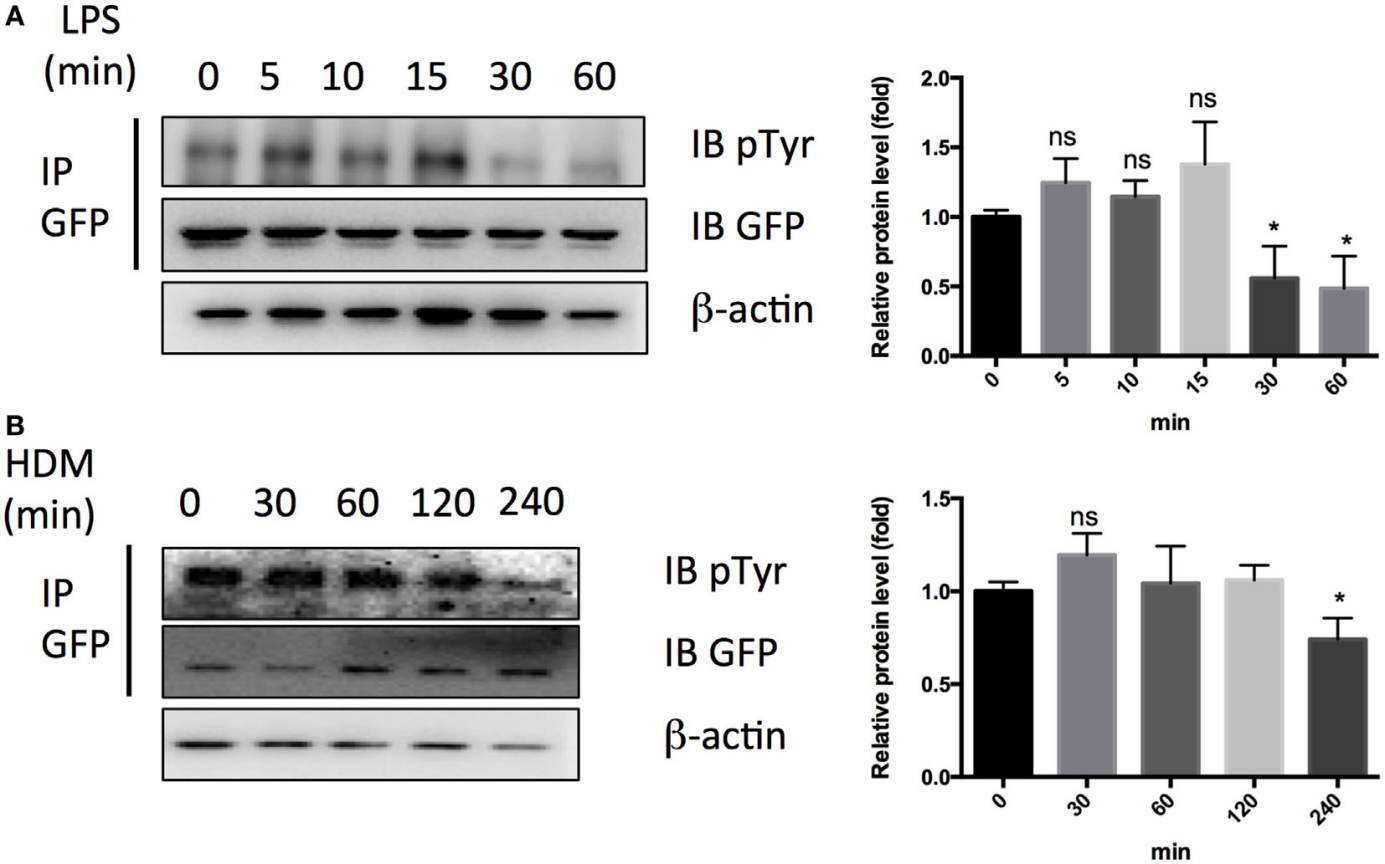
Dephosphorylation of polymerase I and transcript release factor in response to LPS and HDM. **(A)** Whole cell lysates from HBE cell treated with LPS (1 ng/µl) were immunoprecipitated by GFP antibody followed by SDS-PAGE and blotted with indicated antibodies. Quantitative analysis of pTyr/GFP ratio is shown in right panel. **(B)** Whole cell lysates from HBE cell treated with LPS (1 ng/µl) were immunoprecipitated by GFP antibody followed by SDS-PAGE and blotted with indicated antibodies. Quantitative analysis of pTyr/GFP ratio is shown in right panel. *p < 0.05, **p < 0.01, ***p < 0.001, as determined by Student’s *t*-test.

Finally, we checked the effects of LPS or HDM on tyrosine phosphorylation of PTRF and observed an obvious tyrosine dephosphorylation of PTRF in response to LPS treatment for 30 min and HDM treatment for 4 h (Figures 7A,B). Taken together, phosphorylation of PTRF critically regulates its cellular localization and subsequent IL33 release.

## Discussion

Polymerase I and transcript release factor is the most highly expressed and most intensively studied member of cavin family. PTRF anchors caveolin-1 to the cytoskeleton to stabilize caveolae (17). Caveolae are 50–100 µm cup/Ω-shaped stable lipid raft regions and are abundantly presented in vascular endothelial cells, adipocytes, smooth muscle cells, and fibroblasts (18). These multifunctional organelles have roles in endocytosis, cholesterol homeostasis, mechanosensing, and signal transduction (19). Our TAP and Co-IP results have confirmed the interaction of IL-33 and PTRF, we first suspected whether PTRF would mediate the release of IL-33. However, we observed that partial loss of PTRF led to an increased level of IL-33 in BALF in OVA-induced asthma model, which led to excessive type 2 immune responses. Furthermore, this enhanced release of IL-33 was repeatable *in vitro* when PTRF was knocked down. Thus, in contrast to our assumption, PTRF does not mediate the release of IL-33, but controls the release of IL-33. Moreover, G6PD measurement showed that knockdown of PTRF did not lead to cell necrosis, indicating that the increased IL-33 in supernatant was released from living cells, and that PTRF may be an important factor controlling IL-33 release from living cells.

Although the predicted mass is 43 kDa, PTRF is frequently detected at 50–60 kDa following SDS/PAGE. Such a shift is characteristic of multiple posttranslational modifications, and PTRF has been detected in phosphorylated (11), SUMOylated, and ubiquitinated (20). Phosphorylation critically regulates PTRF function. In adipose tissue, PTRF regulates the hydrolysis of triacylglycerols during periods of fasting. Starvation or high-level intracellular cAMP induces PTRF phosphorylation in a PKA (protein kinase A)-dependent manner (21) at multiple sites, including Ser42, Thr304, and Ser368. Upon re-feeding or treatment with insulin, PTRF is phosphorylated at Tyr14, Tyr158, Tyr310, and Tyr318, resulting in translocation to the cytoplasm (21). Here, we report that LPS or HDM stimulation dephosphorylated PTRF majorly at Tyr158 site in 16HBE, resulting in translocation of PTRF to nucleus. Moreover, over-expression of dephosphorylated PTRF efficiently sequestered IL-33 in nucleus even with LPS or HDM stimulation. These results suggest that PTRF can control IL-33 subcellular location under different circumstances, not only allergen exposure, but also bacterial infection.

IL-33 is essential for pathogen defense. IL-33-deficient mice were not able to effectively clear worms due to a selective defect in ILC2-derived IL-13 (22). IL-33 is also involved in tissue repair after viral infection. After infection with influenza virus, IL-33/ST2 signaling was involved in ILC2-dependent restoration of airway epithelial integrity (23). Moreover, Treg cells produce amphiregulin to prevent lung damage in response of alarmin IL-33 after infection with influenza virus (24).

However, excessive IL-33 led to allergic inflammation, including airway allergic inflammation and food-induced anaphylaxis. Chu et al. showed that IL-33 signaling was required for Th2 priming to HDM and peanut, and these effects were associated with an ability of IL-33 to increase OX40L levels on DCs and to expand ILC2s (25). Eosinophilic lung inflammation and Th2 cell differentiation were all found to be impaired in IL-33-deficient mice (3).

Alarmin full-length IL-33 is first released by epithelial cells. This alarmin IL-33 binds to cells expressing IL-1R3 and IL-1R4, including mast cells, DCs, macrophages, group 2 innate lymphoid cells, and memory T cells (10). When mast cells are stimulated by IL-33, they release a Th2-type profile cytokines, including IL-4 and IL-5. In addition, proteases released by activated mast cells cleave full-length IL-33, resulting in a rapid enhancement of IL-33 biological activity (26). Furthermore, the activated mast cells actively release IL-33 to substitute the alarmin IL-33 (27).

Although mast cells are the main source of IL-33, the small amount of IL-33 released by epithelial cells triggers the whole immune response. Thus, it is meaningful to investigate whether the epithelial cells can actively release IL-33 without necrosis and how it is regulated. Our results showed that 16HBE could actively release IL-33 without cell necrosis in response to LPS or HDM, and it is regulated by PTRF.

Previous studies indicated that subcellular location of IL-33 is critical to its function. Gordon et al. (15) studied airway epithelial cells of asthmatics and found that in non-exacerbating asthmatics, the expression of one alternative splicing of the IL-33 transcript (the deletion of exons 3 and 4, Δ exon 3,4) is strongly associated with airway type 2 inflammation, whereas full-length IL-33 is not. This alternative splicing of the IL-33 transcript conferred cytoplasmic localization and facilitates extracellular secretion. Bessa et al. found that altered subcellular localization of IL-33 led to non-resolving lethal inflammation. In their study, IL-33 only with C-terminal had a cytoplasmic location and led to a spontaneous non-resolving fatal inflammation (28). In contrast, IL-33 in nucleus showed some protective roles in epithelial cell repair (29). We observed that with the sequestration of IL-33 in nucleus by dephosphorylated PTRF, the release of IL-33 to supernatants sharply decreased. On the other hand, knockdown of PTRF led to an increased level of IL-33 in supernatants. These results provide more evidence that the subcellular location of IL-33 is critical for its extracellular secretion.

To our knowledge, this is the first study reporting the dephosphorylation of PTRF on response of LPS or HDM and the function of PTRF to sequestrate IL-33. The dephosphorylation of PTRF could be looked as a defense reaction to LPS or HDM, which fine-tuned the release of alarmin IL-33. Controlled release of IL-33 is critical for appropriate immune response. When PTRF was lost, IL-33 would be excessively released, resulting in allergic airway inflammation. However, total lost of PTRF is rare in nature, the regulation of PTRF phosphorylation is more meaningful. Impaired regulation of PTRF phosphorylation may lead to insufficient pathogen defense (excessive dephosphorylation) or allergic inflammation (insufficient dephosphorylation).

Our study has some limitations. First, we used PTRF^+/−^ mice instead of PTRF^−/−^ mice to conduct experiments. This is mainly due to the low birth rate of PTRF^−/−^ mouse. Moreover, PTRF^+/−^ mouse had a very low protein level of PTRF in lung (Figure S3 in Supplementary Material) and showed an obvious phenotype in asthma model. Our results made us sure of the critical role of PTRF during asthma exacerbation. Second, it will be more persuasive to use IL-33 knockout mice or IL-33 neutralizing antibody to further determine the importance of IL-33 signal under PTRF loss circumstance. Third, PTRF has several tyrosine phosphorylation sites. In our study, we only checked the function of Tyr158 phosphorylation of PTRF. Considering that the phosphorylation status of Tyr158 significantly affects the interaction of IL-33 and PTRF, we believe Tyr158 should be the most important phosphorylation site of PTRF.

**Figure 8:**
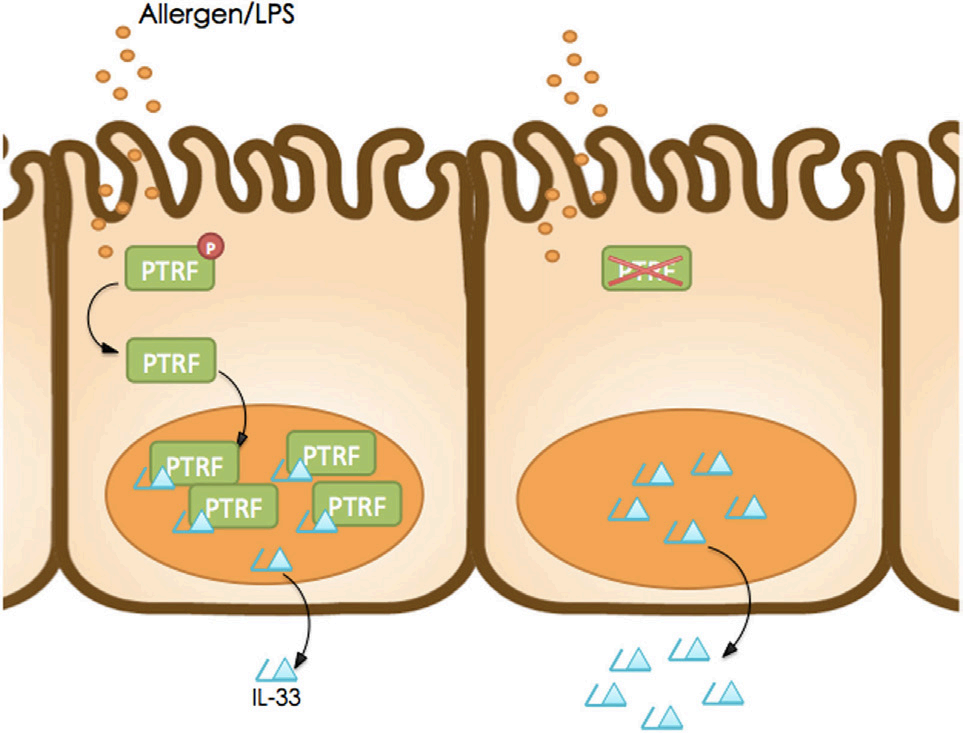
Working model. Dephosphorylation of polymerase I and transcript release factor (PTRF) in response to LPS or HDM stimulation leads to nucleus translocation of PTRF and then controls IL-33 release.

In summary, our study reveals a new mechanism of IL-33 release by living cells, which is controlled by PTRF (Figure 8). Dephosphorylation of PTRF in response to LPS or HDM stimulation leads to nucleus translocation of PTRF, and then controls IL-33 release. This controlled release of IL-33 eventually prevents the development of asthma exacerbation, and thus PTRF is a potential drug target for future anti-asthma immunotherapy.

## Materials and Methods

### Mice

The PTRF^+/−^ mice (B6.129S6-Ptrftm1Pfp/J) were kindly given by Professor Kan Liao. Female PTRF^+/−^ mice were used at 6- to 8-month old. Since that PTRF^−/−^ mouse had a very low birth rate, and PTRF^+/−^ mouse had a very low protein level of PTRF in lung (Figure S3 in Supplementary Material), we used PTRF^+/−^ mice to conduct the experiments. Randomization and blinding strategy was used whenever possible. All mice were maintained under specific pathogen-free conditions. All mouse experiments followed protocols approved by the Institutional Animal Care and Use Committee in Institut Pasteur of Shanghai.

### Asthma Model

The mice received an intraperitoneal injection of 100 µg ovalbumin (OVA; Sigma Aldrich; Merck KGaA) and 2 mg alum (Sigma Aldrich; Merck KGaA) in PBS on days 0, 7, and 14. On days 21–27, the mice were challenge with aerosolized 1% OVA in PBS for 30 min. Control animals received PBS intraperitoneally with alum on days 0, 7, and 14 and were challenged with aerosolized PBS on days 21–27.

Airway hyperresponsiveness was assessed by measuring changes of dynamic lung compliance (Cdyn) in response to increasing doses (0–4 mg/ml) of acetylcholine (Ach; Shanghai Mengry Biotechnology Co., Ltd., Shanghai, China) injected into the tail intravenously in anesthetized and ventilated mice. AHR was assessed using the AniRes2005 animal lung function analysis system (Beijing Bestlab High-Tech Co., Ltd., Beijing, China) 24 h after the last OVA challenge. HE staining and PAS staining were performed as previously described (30).

### Antibodies

CD4-Percp (eBioscience, 46-0041), IL-4-APC (MACS, 130-103-002), IL-5-PE (eBioscience, 12-7052), and IL-13-PE Cy7 (eBioscience, 25-7133) were used for flow cytometry. Anti-IL-33 (Abcam, Ab54385) was used for immunofluorescence of mice lung tissues. Anti-PTRF (CST, 46379), anti-Flag (Sigma-Aldrich, M2), anti-HA (Santa Cruz, sc-7392), anti-GFP (Santa Cruz, sc-9996), anti-pTyr (Santa Cruz, sc-7020), anti-β-Actin (Sungene biotech, KM9001T), anti-α-Tubulin (Sungen biotech, KM9003T), and anti-Lamin B (CST, 13435) were used for immunoblotting.

### Flow Cytometry

To enrich lymphocytes from the lung, mice were transcardially perfused with heparin (10 U ml^−1^) in PBS under anesthesia. Tissues were excised independently of lymph nodes and were incubated for 1 h at 37°C in Collagenase D (1 mg ml^−1^; Roche), DNase (20 mg ml^−1^, Roche), and 0.2% bovine serum albumin in RPMI 1640 medium. Digested tissue was strained through cell strainers. Lymphocytes were further separated by centrifugation through a 40–70% percoll gradient and then subjected to Flow Cytometry Analysis.

For analysis of surface markers, cells were stained in PBS containing 2% fetal bovine serum (FBS) with antibodies as indicated. To determine cytokine expression, cells were stimulated with phorbol12-myristate 13-acetate (50 ng ml^−1^), ionomycin (1 mM), Golgi Stop, and Golgi Plug for 4 h. At the end of stimulation, cells were stained with fixable viability dye eFluor 780 and the indicated antibodies according to the manufacturer’s instructions (eBioscience).

### Cell Culture and Transient Transfection

16HBE cells were cultured in DMEM supplemented with 10% FBS (GIBCO), 1% GlutaMax (GIBCO), 1% sodium pyruvate (GIBCO), and 1% Pen/Strep (GIBCO), and tested for mycoplasma contamination before use. The transfection was performed with Lipoplus (SAGE) following manufacture’s protocol. Experiments were conducted 48 h after transfection. For PTRF knockdown assay, the following shRNA sequences were used:

shPTRF-1 3′-ccggcctggagaagacgcgcctcaactcgagttgaggcgcgtcttctccaggtttttg-5′;
shPTRF-2 3′-ccggcgcgagacaagttgcgcaaatctcgagatttgcgcaacttgtctcgcgtttttg-5′;
sh PTRF-3 3′-ccgggtggaggttgaggaggttattctcgagaataacctcctcaacctccactttttg-5′.

### RNA Preparation and Immunoblotting

Total RNA was extracted using TRIzol reagent (Invitrogen). cDNA was synthesized using a reverse transcriptase kit (TaKaRa, Japan), followed by qRT-PCR analysis (SYBR Green; TaKaRa).

Cells were washed with ice-cold PBS and lysed on ice for 30 min in RIPA buffer (50 mM Tris–HCl, pH 7.5; 135 mM NaCl; 1% NP-40; 0.5% sodium DOC; 1 mM EDTA; 10% glycerol) containing protease inhibitor (1:100, P8340; Sigma-Aldrich), 1 mM NaF, and 1 mM PMSF. Cell lysates were cleared by centrifugation, and supernatants were immunoprecipitated with the appropriate antibodies (Abs, 1 mg ml^−1^) using protein A/G-agarose beads at 4°C. Samples were then used for immunoblotting analysis with indicated antibodies.

### Immunofluorescence

For immunofluorescence imaging, cells were fixed with 4% paraformaldehyde in PBS and permeated with 0.1% Triton X-100 and 3% bovine serum albumin in PBS. The cells were then incubated with primary antibody and fluorescein-conjugated secondary antibody and photographed by Leica SP8 laser scanning confocal microscope. For cells expressing eGFP-tagged protein or RFP-tagged protein, the paraformaldehyde fixed cells were visualized directly by confocal microscope.

Mice lung tissues were embedded in paraffin with isector molds and regular paraffin molds, as previously described (2). 3 µm sections were stained for IL-33 using anti-IL33 antibody (Abcam, Ab54385) or IgG control. Nuclei were visualized with DAPI (1:500). Tissues were then photographed by Leica SP8 laser scanning confocal microscope.

### ELISA and G6PD Release Assay

Concentrations of cytokines in the BALF and culture supernatant were measured by ELISA, according to the protocols of each ELISA kit (IL-4 Mouse ELISA Kit, Raybio, ELM-IL4; IL-5 Mouse ELISA kit, eBioscience, 88-7054; IL-13 Mouse ELISA Kit, Invitrogen, KMC2221; IL-33 Mouse ELISA Kit, Affymetrix, 88-7333-22). G6PD release assay were performed by using the Vibrant Cytotoxicity Assay Kit (Molecular Probes, USA).

### Statistical Analysis

*p-*Values were calculated with Student’s *t*-test or analysis of variance (GraphPad Prism) as specified in figure legends. All data represent means ± SD. *p < 0.05, **p < 0.01, ***p < 0.001, as determined by two-tailed, unpaired Student’s *t*-test. NS, not significant. Sample sizes were designed with adequate power according to the literature. Randomization and blinding strategy was used whenever possible.

## Ethics Statement

This study was carried out in accordance with the recommendations of the Institutional Animal Care and Use Committee in Institut Pasteur of Shanghai. The protocol was approved by the Institutional Animal Care and Use Committee in Institut Pasteur of Shanghai.

## Author Contributions

YN designed and performed experiments and wrote the manuscript; JH and XH contributed to cellular experiments; WD and YY contributed to the technical support; TC, YL, and ZW contributed to animal experiments; YL and FZ contributed to scientific discussion, SW, RL, and DL contributed to writing and editing the manuscript; KL, BL, and GS designed experiments, contributed to writing the manuscript, and provided overall direction.

## Conflict of Interest Statement

The authors declare that the research was conducted in the absence of any commercial or financial relationships that could be construed as a potential conflict of interest.

## Supplementary Material

Figure S1 IL-33 interacts with polymerase I and transcript release factor (PTRF). Whole cell lysates from Flag-IL-33 and HA-PTRF transfected HBE cells were immunoprecipitated with anti-HA or anti-Flag antibody followed by SDS-PAGE and blotted with indicated antibodies.

Figure S2 Partial loss of polymerase I and transcript release factor (PTRF) does not affect airway inflammation during sensitization phase. Mice were intraperitoneally injected with phosphate-buffered saline or ovalbumin on days 0, 7, and 14 and then sacrificed on day 15. **(A)** Representative images of hematoxylin and eosin staining of lung tissue (upper panel), and periodic acid-schiff stain (lower panel). **(B)** Percentages of IL-4^+^, IL-5^+^, IL-13^+^ CD4^+^ T cells from lung, spleen, and peripheral lymph nodes of WT and PTRF^+/−^ littermates. **(C)** IL-4, IL-5, and IL-13 levels in culture supernatant of lymphocytes from lung, spleen, and peripheral lymph nodes of WT and PTRF^+/−^ littermates. Results are pooled data from four independent experiments (mean ± SEM of *n* = 4 mice in each group).

Figure S3 Protein level of polymerase I and transcript release factor in lungs.
