# Supplementary figures and images for: Dephosphorylated Polymerase I and Transcript Release Factor Prevents Allergic Asthma Exacerbations by Limiting IL-33 Release
Source: Front Immunol. 2018 Jun 21;9:1422. doi: 10.3389/fimmu.2018.01422 (PMC6021487; doi:10.3389/fimmu.2018.01422)

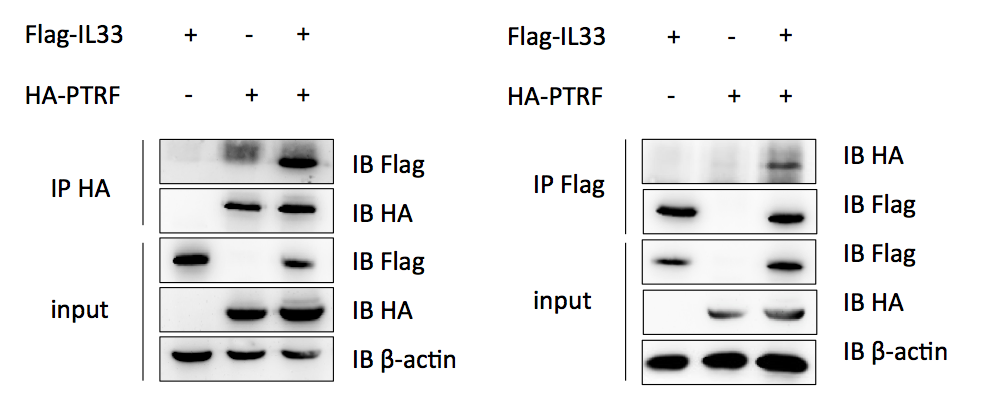

Supplement: Figure S1 — IL-33 interacts with polymerase I and transcript release factor (PTRF). Whole cell lysates from Flag-IL-33 and HA-PTRF transfected HBE cells were immunoprecipitated with anti-HA or anti-Flag antibody followed by SDS-PAGE and blotted with indicated antibodies. [file image_1.tiff]

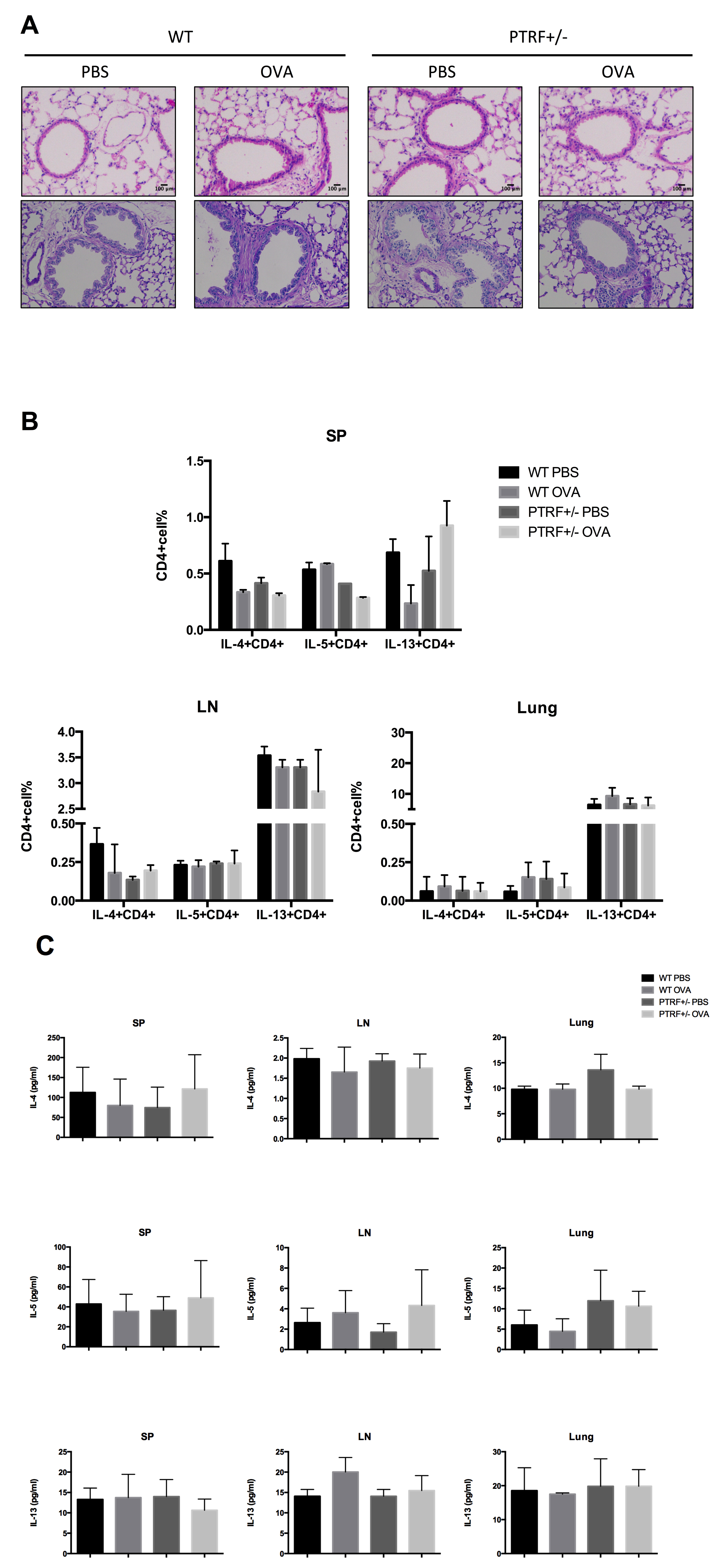

Supplement: Figure S2 — Partial loss of polymerase I and transcript release factor (PTRF) does not affect airway inflammation during sensitization phase. Mice were intraperitoneally injected with phosphate-buffered saline or ovalbumin on days 0, 7, and 14 and then sacrificed on day 15. (A) Representative images of hematoxylin and eosin staining of lung tissue (upper panel), and periodic acid-schiff stain (lower panel). (B) Percentages of IL-4+, IL-5+, IL-13+ CD4+ T cells from lung, spleen, and peripheral lymph nodes of WT and PTRF+/− littermates. (C) IL-4, IL-5, and IL-13 levels in culture supernatant of lymphocytes from lung, spleen, and peripheral lymph nodes of WT and PTRF+/− littermates. Results are pooled data from four independent experiments (mean ± SEM of n = 4 mice in each group). [file image_2.tiff]

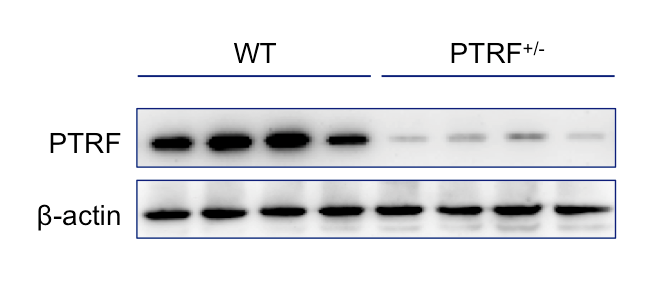

Supplement: Figure S3 — Protein level of polymerase I and transcript release factor in lungs. [file image_3.tiff]
